# Misdiagnosis of narcolepsy

**DOI:** 10.1007/s11325-016-1365-5

**Published:** 2016-06-23

**Authors:** Laura Dunne, Pallavi Patel, Emily L Maschauer, Ian Morrison, Renata L Riha

**Affiliations:** 1 Sleep Research Unit, Department of Sleep Medicine, University of Edinburgh, Royal Infirmary of Edinburgh, 51 Little France Crescent, Edinburgh, EH16 4SA UK; 2 Department of Neurology, University of Dundee, Ninewells Hospital, Dundee, DD1 9SY UK

**Keywords:** Narcolepsy, Diagnosis, Misdiagnosis, Cataplexy, MSLT

## Abstract

**Background:**

Narcolepsy is a chronic primary sleep disorder, characterized by excessive daytime sleepiness and sleep dysfunction with or without cataplexy. Narcolepsy is uncommon, with a low prevalence rate which makes it difficult to diagnose definitively without a complex series of tests and a detailed history. The aim of this study was to review patients referred to a tertiary sleep centre who had been labelled with a diagnosis of narcolepsy prior to referral in order to assess if the diagnosis was accurate, and if not, to determine the cause of diagnostic misattribution.

**Methods:**

All patients seen at a sleep centre from 2007–2013 (*n* = 551) who underwent detailed objective testing including an MSLT PSG, as well as wearing an actigraphy watch and completing a sleep diary for 2 weeks, were assessed for a pre-referral and final diagnosis of narcolepsy.

**Results:**

Of the 41 directly referred patients with a diagnostic label of narcolepsy, 19 (46 %) were subsequently confirmed to have narcolepsy on objective testing and assessment by a sleep physician using ICSD-2 criteria.

**Conclusions:**

The diagnosis of narcolepsy was incorrectly attributed to almost 50 % of patients labelled with a diagnosis of narcolepsy who were referred for further opinion by a variety of specialists and generalists. Accurate diagnosis of narcolepsy is critical for many reasons, such as the impact it has on quality of life, driving, employment, insurance and pregnancy in women as well as medication management.

## Introduction

Narcolepsy is a chronic primary sleep disorder, characterized by excessive daytime sleepiness (EDS) with symptoms of rapid eye movement (REM) and sleep dysfunction (i.e. sleep paralysis, hypnagogic hallucinations), with or without cataplexy (muscle weakness). Narcolepsy is uncommon and the estimated prevalence ranges from 0.01 to 0.05 % worldwide. [1] The low prevalence means a definitive diagnosis of narcolepsy can be complex and should incorporate a detailed presenting history, assessment of sleep-wake cycles, assessment of sleep deprivation, subjective testing (sleep diary) and objective testing (polysomnography (PSG), actigraphy, multiple sleep latency testing (MSLT) including drug-testing of urine, human leukocyte antigen (HLA) typing and lumbar puncture to measure CSF hypocretin-1 (orexin) levels) [2].

Even with the use of the International Classification of Sleep Disorders-2 (ICSD-2) [9] and the International Classification of Sleep Disorders-3 (ICSD-3) [10] (since 2014), uncertainty with diagnosis may persist with the most experienced clinicians, given that the symptoms of narcolepsy can often be mimicked by other sleep disorders (e.g. sleep disordered breathing) [11] or overlap with psychiatric symptomology. [8] Patients have been misdiagnosed with narcolepsy on the basis of clinical judgement alone, resulting in inappropriate treatment. [3, 4] The aim of this study was to review patients who had been labelled with a diagnosis of narcolepsy by a variety of specialists and generalists prior to referral to a tertiary sleep centre for a second opinion and/or continued management, as well as to determine the basis for any diagnostic misattribution.

## Methods

A database comprising all patients seen at a tertiary referral sleep centre undergoing MSLT between October 2007 and July 2013 (*n* = 551) was cross-examined. ‘Diagnosis of narcolepsy’ on the referral letter was defined as patients given a diagnosis of ‘narcolepsy’, patients labelled with ‘probable narcolepsy’ (with/without treatment) and patients deemed as ‘possibly’ having narcolepsy provided they had received treatment with stimulant medication prior to referral. All patient referrals came from regional centres that did not have expertise in non-respiratory sleep medicine. Most patients had received little to no assessment or testing for narcolepsy before presenting at the sleep clinic (very few had a limited study or PSG, but none had an MSLT). The ICSD-2 [9] was used to determine criteria for the narcolepsy and cataplexy diagnosis. All patients were subsequently reviewed by one of two sleep specialists in the clinic. Given the frequency of comorbidity of other sleep disorders in the narcolepsy population (e.g. obstructive sleep apnoea), all patients underwent an MSLT and PSG and wore an actigraphy watch and kept a sleep diary for 2 weeks prior to in-lab testing. All patients underwent further assessment in the sleep clinic to ensure that symptoms were more likely as a result of narcolepsy and not due to other sleep disorders.

Sole reliance for diagnosis was not placed on the MSLT. The MSLT should not be used as a diagnostic tool in isolation, [15] due to its limited test-retest reliability, sometimes yielding misleading or false positive or negative results, [12] and its inadequate exclusion of other sleep disorders (e.g. OSAHS). [3] Arand et al. [15] recommend improving the reliability of the MSLT by combining it with the results from a PSG and obtaining a suitable history of the patient. The sleep specialists reviewed the referrals in addition to obtaining detailed history directly from the patient and acquiring information from objective test results from actigraphy over 2 weeks, PSG followed by MSLT, a urine drug screen, as well as a subjective 2-week sleep diary. Difficult cases were discussed at multi-disciplinary meetings and diagnosis was achieved by consensus and further review. Investigation and scoring of sleep data were conducted in a blinded manner; technicians who scored the data were not involved with history taking nor allowed to review the patient files to prevent any bias in scoring. Specific ethical approval was not considered necessary, as the Ethics Advisory Committee in Edinburgh does not require approval for case series.

Data were gathered on presenting history, medication, referral/diagnosis, objective investigations, co-morbidities and anthropometrics. Analysis, using standard statistical techniques, was undertaken with SPSS (IBM, v.19). Significance was taken at *p* ≤ 0.05, and all tests were two-tailed.

## Results

Of the 41 patients referred with a ‘diagnosis of narcolepsy’ over the 6-year period, only 19 (46 %) were confirmed to have narcolepsy after further assessment and objective testing. The source of the initial diagnosis in the majority (*n* = 18) was a general practitioner; in the remainder, diagnoses had been made by a neurologist (*n* = 8), a regional sleep clinic (*n* = 9), or respiratory physician (*n* = 6).

Females were significantly more likely to be diagnosed with narcolepsy than males (15 vs. 7; *p* = 0.002).

A history of cataplexy was present in 84 % of the confirmed narcoleptic patients and 27 % of the pre-reported non-narcoleptics (*p* < 0.001). EDS was measured subjectively by the Epworth sleepiness scale, [5] and the results were equivalent between groups (15.15 ± 5.43 in narcoleptics vs. 15.50 ± 4.53 in non-narcoleptics, *p* = 0.85).

The median sleep onset latency on overnight PSG was lower in patients with confirmed narcolepsy than for patients without confirmed narcolepsy (6.5 ± 7.86, *p* = 0.005 vs. 21.5 ± 34.63, *p* < 0.0001), as was mean sleep latency on MSLT (3.65 ± 2.95 vs. 10.20 ± 4.86, *p* < 0.0001). All confirmed narcoleptics experienced at least two SOREMPs on the MSLT. Six non-narcoleptics were able to achieve REM sleep on the MLST. Mean REM latency was significantly lower in the confirmed narcoleptics vs. non-narcoleptics (2.7 vs. 7.8 min, *p* = 0.007). The number of sleep onset REM-periods was greater in patients with confirmed narcolepsy compared to those without (3.1 ± 1.1 vs 1.6 ± 0.8, *p* = 0.004). DQB1*0602 alleles were found in 90 % of the confirmed narcolepsy patients, in 40 % of the non-narcoleptic patients (*p* = 0.037) and in the three (16 %) patients who had narcolepsy without cataplexy. Despite the fact that 90–95 % of the narcolepsy population will have a positive HLA for narcolepsy, [13] we used the HLA markers as an additive factor, not the only component, to support the diagnosis of narcolepsy. Three non-narcoleptic patients who had CSF-hypocretin-1 measured had entirely normal levels (>110 pg/ml).

Only three patients without narcolepsy had no discernible explanation for their symptoms. Diagnoses in the remaining 19 were obstructive sleep apnoea syndrome (*n* = 10), depression/anxiety (*n* = 3), sleep deprivation (*n* = 3), irregular sleep/wake cycles (*n* = 2) and parasomnia (*n* = 1) (Table 1). Thus, non-narcoleptics and patients with EDS differed from true narcoleptics determined by the assessment tests described above.

**Table 1:** Demographic and diagnosis information at referral for 41 patients referred with a ‘diagnosis of narcolepsy’

Case	Age	Prior diagnosis	Medication	PSG results	MSLT results	Drug screen^a^	HLA type	Features of cataplexy	Final diagnosis
1	28	Probable Nx	None	SE: 87.6 % SOLmin: 3.4 REML: 0 min AHI: 52.9 events/h	MSL: 2.0 min MREML: 0.3 No. naps with REM: 4	Negative	Blood could not be obtained or patient refused	Present	True Nx
2	26	Probable Nx	None	SE: 84.6 % SOLmin: 2.5 REML: 0.5 min AHI: 10.1 events/h	MSL: 1.9 min MREML: 2.6 No. naps with REM: 4	Negative	DQB1*06:02	Present	True Nx
3	56	Nx	Dipyridamole, ezetimibe, aspirin, perindopril erbumine, bendroflumet-hiazide, co-codamol	SE: 67.3 % SOLmin: 26.0 REML: 52.0 min AHI: 34.6 events/h	MSL: 8.2 min MREML: 11.0 No. naps with REM: 1	Negative	Negative	Absent	OSAHS
4	55	Nx	Aspirin, bisoprolol, clenil modulite, co-dydramol, furosemide, nitroglycerin, nicorandil, ramipril, salbutamol, simvastatin	SE: 51.5 % SOLmin: 161.0 REML: 95.0 min AHI: 17.8 events/h	MSL: 18.0 min MREML: 0 No. naps with REM: 0	Negative	Negative	Absent	Depression/ anxiety
5	65	Probable Nx	None	SE: 73.4 % SOLmin: 38.0 REML: 45.5 min AHI: 13.6 events/h	MSL: 15.5 min MREML: 0 No. naps with REM: 0	Negative	Negative	Absent	OSAHS
6	37	Nx	None	SE: 88.2 % SOLmin: 9.0 REML: 59.0 min AHI: 8.7 events/h	MSL: 11.6 min MREML: 0 No. naps with REM: 0	Urine could not be obtained or patient refused	DQB1*0603/ 08/14, HLA-DQA1*0103	Present	Depression/ anxiety
7	31	Nx	None	SE: 85.1 % SOLmin: 6.0 REML: 1.0 min AHI: 10.7 events/h	MSL: 4.4 min MREML: 1.0 No. naps with REM: 3	Negative	DQB1*06:02	Absent	True Nx
8	31	Probable Nx	Lansoprazole, thyroxine	SE: 83.0 % SOLmin: 26.5 REML: 95.5 min AHI: 23.4 events/h	MSL: 3.9 min MREML: 13.3 No. naps with REM: 2	Negative	DQB1*0602	Present	Sleep deprivation
9	23	Probable Nx	Salbutamol, oral contraceptive, betamethasone cream, beclomethasonedipropionate inhaler,	SE: 93.0 % SOLmin: 6.0 REML: 57.0 min AHI: 8.2 events/h	MSL: 11.3 min MREML: 0 No. naps with REM: 0	Negative	Blood could not be obtained or patient refused	Absent	True Nx
10	65	Nx	Nitroglycerin, co-codamol, frusemide, telmisartan, rosuvastatin, cordarone, warfarin	SE: 52.1 % SOLmin: 1.5 REML: 223.5 min AHI: 25.6 events/h	MSL: 8.0 min MREML: 0 No. naps with REM: 0	Negative	Positive but type not recorded	Absent	OSAHS
11	50	Probable Nx	Clonidine	SE: 68.2 % SOLmin: 70.0 REML: 322.0 min AHI: 10.3 events/h	MSL: 14.0 min MREML: 0 No. naps with REM: 0	Positive	Blood could not be obtained or patient refused	Present	Irregular sleep
12	51	Nx	None	SE: 85.3 % SOLmin: 6.5 REML: 75.0 min AHI: 10.9 events/h	MSL: 1.8 min MREML: 6.3 No. naps with REM: 3	Negative	DQB1*06, DRB1*15, DQA1*01	Present	True Nx
13	30	Probable Nx	None	SE: 81.3 % SOLmin: 16.5 REML: 134.0 min AHI: 19.2 events/h	MSL: 3.4 min MREML: 3.4 No. naps with REM: 4	Negative	DQB1*06, DRB1*15, DQA1*01	Present	True Nx
14	29	Nx	Pemoline, clomipramine, fluoxetine, cetirizine, selegiline, homeopathic opium, oral contraceptive	SE: 91.5 % SOLmin: 2.5 REML: 61.5 min AHI: 1.0 events/h	MSL: 0.8 min MREML: 0.3 No. naps with REM: 4	Negative	Blood could not be obtained or patient refused	Present	True Nx
15	48	Probable Nx	Thyroxine, cetirizine	SE: 73.6 % SOLmin: 7.5 REML: 0 min AHI: 34.3 events/h	MSL: 2.1 min MREML: 1.0 No. naps with REM: 4	Negative	Blood could not be obtained or patient refused	Present	True Nx
16	30	Probable Nx	Oral contraceptive	SE: 88.4 % SOLmin: 30.0 REML: 76.5 min AHI: 4.3 events/h	MSL: 7.5 min MREML: 3.0 No. naps with REM: 1	Urine could not be obtained or patient refused	Blood could not be obtained or patient refused	Present	True Nx
17	34	Nx	None	SE: 0 SOLmin: 0 REML: 0 min AHI: 0 events/h	MSL: 6.5 min MREML: 15.3 No. naps with REM: 2	Negative	Blood could not be obtained or patient refused	Present	True Nx
18	44	Nx	Dipyridamole, esomeprazole, aspirin, simvastatin, quinine	SE: 78.6 % SOLmin: 14.0 REML: 115.0 min AHI: 35.3 events/h	MSL: 17.3 min MREML: 0 No. naps with REM: 0	Negative	Negative	Absent	OSAHS
19	33	Nx	Orlistat	SE: 86.4 % SOLmin: 1.0 REML: 2.5 min AHI: 8.8 events/h	MSL: 0.8 min MREML: 1.0 No. naps with REM: 4	Negative	DQB1*0602	Present	True Nx
20	30	Nx	Melatonin	SE: 71.6 % SOLmin: 27.0 REML: 61.0 min AHI: 10.3 events/h	MSL: 3.8 min MREML: 2.7 No. naps with REM: 3	Negative	DQB1*0602	Absent	Poor sleep hygiene
21	32	Probable Nx	Pramipexole, pregabalin	SE: 81.8 % SOLmin: 21.0 REML: 104.0 min AHI: 9.5 events/h	MSL: 6.1 min MREML: 10.5 No. naps with REM: 1	Positive	Negative	Present	Parasomnia
22	28	Nx	None	SE: 78.5 % SOLmin: 51.0 REML: 32.5 min AHI: 18.7 events/h	MSL: 13.6 min MREML: 0 No. naps with REM: 0	Negative	Blood could not be obtained or patient refused	Absent	OSAHS
23	69	Nx	Zopiclone, trazadone, thyroxine, tramadol, omeprazole	SE: 68.5 % SOLmin: 23.0 REML: 68.0 min AHI: 19.2 events/h	MSL: 1.1 min MREML: 2.5 No. naps with REM: 2	Negative	Positive but type not recorded	Present	True Nx
24	42	Nx	None	SE: 55.5 % SOLmin: 7.5 REML: 89.0 min AHI: 30.5 events/h	MSL: 4.1 min MREML: 1.3 No. naps with REM: 2	Positive	DQB1*0602/14/15/16/19/20/23/24/33	Present	Depression/ anxiety
25	27	Probable Nx	None	SE: 84.0 % SOLmin: 11.5 REML: 0.5 min AHI: 3.7 events/h	MSL: 2.5 min MREML: 3.6 No. naps with REM: 4	Negative	DQB1*0602	Present	True Nx
26	31	Probable Nx	None	SE: 84.6 % SOLmin: 12.5 REML: 192.5 min AHI: 6.2 events/h	MSL: 7.0 min MREML: 0 No. naps with REM: 0	Positive	DQB1*0602	Absent	Sleep deprivation
27	32	Nx	Mazindol	SE: 84.8 % SOLmin: 7.0 REML: 55.5 min AHI: 14.4 events/h	MSL: 2.4 min MREML: 1.3 No. naps with REM: 4	Positive	Blood could not be obtained or patient refused	Present	True Nx
28	42	Probable Nx	Omeprazole	SE: 94.2 % SOLmin: 4.5 REML: 59.0 min AHI: 26.1 events/h	MSL: 5.1 min MREML: 5.5 No. naps with REM: 1	Positive	Negative	Absent	OSAHS
29	30	Nx	Amitriptyline, modafinil	SE: 86.3 % SOLmin: 34.5 REML: 54.0 min AHI: 6.9 events/h	MSL: 7.9 min MREML: 0 No. naps with REM: 0	Urine could not be obtained or patient refused	Negative	Absent	Irregular sleep
30	22	Nx	None	SE: 94.3 % SOLmin: 10.5 REML: 64.0 min AHI: 5.0 events/h	MSL: 7.5 min MREML: 0 No. naps with REM: 0	Negative	Negative	Absent	Sleep deprivation
31	36	Probable Nx	None	SE: 62.3 % SOLmin: 22.0 REML: 46.0 min AHI: 10.0 events/h	MSL: 9.0 min MREML: 0 No. naps with REM: 0	Urine could not be obtained or patient refused	Blood could not be obtained or patient refused	Present	Poor sleep hygiene
32	60	Nx	Lansoprazole, co-codamol, domperidone, modafinil	SE: 64.8 % SOLmin: 17.5 REML: 58.0 min AHI: 24.2 events/h	MSL: 10.4 min MREML: 0 No. naps with REM: 0	Positive	Negative	Absent	OSAHS
33	20	Nx	None	SE: 89.6 % SOLmin: 15.0 REML: 147.0 min AHI: 7.3 events/h	MSL: 5.3 min MREML: 1.0 No. naps with REM: 3	Negative	Negative	Absent	True Nx
34	48	Probable Nx	None	SE: 56.9 % SOLmin: 7.0 REML: 66.0 min AHI: 4.5 events/h	MSL: 4.5 min MREML: 3.0 No. naps with REM: 2	Negative	Blood could not be obtained or patient refused	Present	True Nx
35	36	Nx	None	SE: 57.4 % SOLmin: 3.0 REML: 93.5 min AHI: 38.5 events/h	MSL: 11.1 min MREML: 0 No. naps with REM: 0	Negative	Blood could not be obtained or patient refused	Absent	OSAHS
36	43	Nx	Salbutamol, beclometasone, iron tablets	SE: 68.9 % SOLmin: 7.5 REML: 101.0 min AHI: 108.7 events/h	MSL: 1.8 min MREML: 2.5 No. naps with REM: 1	Negative	DQB1*0602	Present	True Nx
37	57	Probable Nx	None	SE: 70.5 % SOLmin: 0.0 REML: 0 min AHI: 18.5 events/h	MSL: 0.8 min MREML: 0.1 No. naps with REM: 4	Negative	DQB1*0602	Present	True Nx
38	34	Nx	Diclofenac	SE: 77.4 % SOLmin: 11.5 REML: 114.0 min AHI: 2.0 events/h	MSL: 6.9 min MREML: 10.0 No. naps with REM: 1	Negative	DRB1*15, DRB5*01/02, DQB1*06, DQA	Absent	Poor sleep hygiene
39	49	Nx	Olanzapine, aspirin, metformin, atorvastatin, furosemide, diclofenac, co-codamol, levothyroxine, hydrocortisone,	SE: 78.8 % SOLmin: 40.5 REML: 107.0 min AHI: 16.5 events/h	MSL: 15.5 min MREML: 0 No. naps with REM: 0	Negative	Negative	Absent	OSAHS
40	62	Nx	Fentanyl patch, levothyroxine, bendroflumethia-zide, aspirin, amitriptyline, acetaminophen, diazepam, perindopril	SE: 29.8 % SOLmin: 62.0 REML: 0 min AHI: 7.2 events/h	MSL: 20.0 min MREML: 0 No. naps with REM: 0	Negative	Negative	Absent	OSAHS
41	63	Nx	Amitryptiline, gliclazide, metformin, simvastatin	SE: 37.9 % SOLmin: 6.5 REML: 97.0 min AHI: 71.1 events/h	MSL: 8.4 min MREML: 0.8 No. naps with REM: 2	Negative	Negative	Present	True Nx

*Abbreviations*: *PSG* polysomnography, *MSLT* multiple sleep latency test, *HLA* human leukocyte antigen, *Nx* narcolepsy, *SE* sleep efficiency, *REML* rapid eye movement latency, *AHI* apnea hypogea index, *MSL* mean sleep latency, *REM* rapid eye movement, *OSAHS* obstructive sleep apnea hypoventilation syndrome, *SOL* sleep onset latency, *MREML* mean rapid eye movement latency

^a^We routinely screen for drugs of abuse consisting of opiates, amphetamines, cocaine, methadone and benzodiazepines. Cannabinoids are also included

**Fig. 1:**
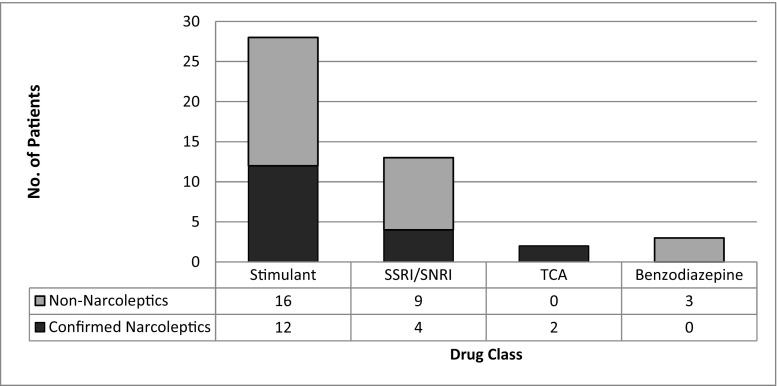
Drug treatment at referral for 41 patients referred with a ‘diagnosis of narcolepsy’. *SSRI* selective serotonin re-uptake inhibitor, *SNRI* serotonin–norepinephrine re-uptake inhibitor, *TCA* tricyclic antidepressant

Prior to referral, 31 patients were prescribed CNS-altering medication (Fig. 1). Of 28 patients receiving stimulants, 16 did not have narcolepsy. The specific drugs used in this latter patient group were modafinil (*n* = 10), dexamphetamine (*n* = 5) and methylphenidate (*n* = 1).

## Discussion

A diagnosis of narcolepsy was incorrectly attributed to almost 50 % of patients referred for further opinion by a variety of specialists and generalists. We are one of the only specialized sleep clinics in the country; therefore, the high number of innacurately diagnosed patients could result from referral bias. Easily recognized and accurately diagnosed cases of narcolepsy which do not require clarification would possibly not be referred to the sleep clinic for validation and we may be receiving only cases that pose difficult diagnostic conundrums for referring doctors. The only significant difference between narcoleptics and non-narcoleptics on presentation was a history of cataplexy, but this was not exclusive to narcoleptics. [6] Objective testing using PSG and MSLT was discriminatory between groups, in keeping with previous studies. [7].

Accurate diagnosis of narcolepsy is critical for many reasons, such as the impact it has on quality of life, driving, employment, insurance and implications regarding pregnancy in women. Treatment involves the use of controlled (e.g. amphetamines) and expensive (e.g. sodium oxybate) drugs which should not be prescribed in error, as there are not inconsiderable potential side effects (e.g. nausea, weight loss, psychiatric complications), tolerance and possible addiction issues. [1] Health service costs resulting from inappropriate treatment are also an important consideration. [1, 3, 6] Furthermore, several countries require a diagnosis of a medical condition in order to approve prescription medication. With the use of the Internet and increasing information disseminated on television programmes, many people self-diagnose, presenting at clinics with highly educated information and an excellent history of the disorder. This can be problematic because drug-seeking individuals may present with fabricated narcolepsy symptoms in order to obtain stimulant medications. [14] The use of illegal substances can also influence the results of MSLT and urine drug screening remains of great importance in accurate diagnosis and treatment of adults and children. [2, 16].

The study sample of 41 patients over a 6-year time period—with 19 patients correctly diagnosed with narcolepsy—might be considered small. However, the figures are high when taken in the context of narcolepsy being an uncommon disorder with a prevalence of 0.01 % in the general population. A limitation to our study is that we were unable to calculate the degree of certainty of diagnosis and if the source of initial diagnosis had an impact on the confirmation rate due to the small sample size. In an age when access to specialized diagnostic facilities is unrestricted, it is of concern that such diagnostic inaccuracy continues to occur. It is hoped that the recent publication of the ICSD-3 with its detailed instructions on diagnosing narcolepsy will further reduce misdiagnosis by giving a clearer and more accurate description for providers to use.
